# Uterine Diseases

**Published:** 1868

**Authors:** J. G. Westmoreland

**Affiliations:** Professor of Materia Medica and Therapeutics in Atlanta Medical College


### ARTICLE II.

UTERINE DISEASES. By J. G. WESTMORELAND, M. D., Professor of Materia Medica and Therapeutics in Atlanta Medical College.

Perhaps no organ in the body is so prolific of disease, and none has such varied and mysterious symptoms, as the uterus. This is not remarkable, when it is considered physiologically. In addition to the monthly changes which regularly occur, causing plethora to the extent, sometimes, of actual engorgement, it is the natural receptacle, source of nourishment, and place of development for the impregnated ovum. The sudden and vast changes in the amount of blood sent to it in the performance of these functions, and the time of rest which ensues after their accomplishment, require the strictest hygienic measures to prevent that degree of congestion from which generally arise the various forms of local disease to which the womb is subject.

That uterine affections should be treated unprofitably to the patient and unsatisfactorily to the physician, cannot be wondered at, when it is known how very frequently we have an entire misconception of the true pathological state of the womb itself. Functional disturbance is attributed too often to external causes, and to the various forms of general derangement of the system, unconnected directly with this organ. While it is true that, in common with other organs, the uterus fails to perform its functions when the great general influences derived from the nervous and circulatory systems are deranged, before any organic disease exists, yet, in a large majority of cases, this difficulty gives no lasting trouble, unless structural changes of a more or less permanent nature exist in the womb. A thorough acquaintance with the nature of organic diseases of the uterus, and their effects upon the nervous system, is necessary in order to detect in the multiform nervous symptoms which result, the existence of uterine derangement as their cause. These symptoms are often exhibited exclusively in organs perfectly free from structural disease, but whose functions are temporarily disturbed by the reflex nervous impressions upon them on account of the pathological condition of the womb. Moreover, these symptoms are often deceptive as to the actual functional derangement which they seem to indicate. The state of the nervous system thus induced leads to the development of symptoms which are mistaken for those of indigestion, imperfect respiration and cardiac debility, when, in fact, these functions are perfectly performed. Days, months, and even years of treatment have been expended thus upon a healthy organ, on account of the manifestation of symptoms in them which seem to demand treatment. Symptoms resembling those of indigestion, such as pain and fulness of the epigastrum, with eructations, &c., are frequently met with in chronic uterine disease, and are generally found persistent when remedies are directed alone to the stomach. The same may be said of the sensations which are attributed to, at least, functional disease of the heart, when no such disease actually exists.

From these remarks it must not be inferred that, in our opinion, patients of this description dissemble in their manifestations of suffering. Far from it. Our sympathies are always aroused for such unfortunate sufferers. And though no *permanent* relief is obtained by remedies addressed to this state of the nervous system, yet that abatement, so important sometimes to the patient, is secured temporarily. All such manifestations from reflex nervous disturbance in the female are included under the general term *Hysteria*—a term applied to them by ancient medical writers, who attributed them, and properly we think, to uterine disturbance.

We would not be understood to advocate the opinion that the functions of organs remote from the uterus are not sometimes actually deranged from this cause, and even permanent disease in them produced. Such, we have every reason to believe, is frequently the case. With such a constant state of reflex nervous disturbance, the organs thus failing to receive the ordinary healthy nervous supply must, of necessity, suffer at some time functional, and even organic disturbance. The peculiar phases assumed in this form of nervous disturbances, as well as many of the natural phenomena connected with the nervous system, are, in the present state of science, inscrutable. Why it is that in chronic disease of the uterus the reflex influence from the local irritation should affect that portion of the cerebro-spinal centres, situated in the dorsal spine, more prominently than other parts of the cord, has not been satisfactorily explained; and yet the tenderness at this portion of the spinal column, and the manifestation of nervous derangement more frequently in the organs supplied with nerves from this part of the cord, prove conclusively this fact. We would naturally expect to find that portion of the cord most affected by transmission of the local irritation, from which arise the nerves supplying the uterus, yet the above facts, and the frequent absence of such symptoms connected with the lumbar and sacral regions would lead to a different conclusion.

After the consideration of these mysterious developments, the most important matter connected with this subject, and for the discussion of which this article was more particularly intended, is the nature and treatment of the various forms of non-malignant uterine chronic disease to which the organ is liable. We have already stated that disturbance, purely functional, is much less frequently met with than is by some supposed; and that such, when in existence, readily subsides when the irregularities in the governing systems of the body upon which it depends is corrected, we think equally certain. If, however, as before intimated, irregularities of the nervous or circulatory system are suffered to remain till structural changes occur in the uterus, the latter will continue and keep up the functional disturbance, though the first causes may abate. It would seem reasonable to conclude that engorgement is the most frequent primary disturbance in uterine disease. To a want of vitality, however, existing either as the cause or result of an anæmic condition of the womb, doubtless chronic lesions are sometimes due.

We have alluded to the unfortunate mistake sometimes made in treating the results of uterine disease as manifested in the various forms of nervous derangement, with the view of permanent relief. No less so is that directed to the results as exhibited in the womb itself. Various forms of functional disturbance and mal-positions of the uterus result from organic or structural changes. Dysmenorrhœa, suppressed menstruation, &c., are some of the effects of uterine disease upon the function of the organ, and are improperly classed, and for years unsuccessfully treated, as uterine disease by name. The same may be said of the various forms of displacement, &c., of the womb. Prolapsus, the different horizontal variations, and leucorrhœa are also the result or effects of disease, but are often recognized and treated as special disorders, to the utter neglect of the structural disturbance upon which they depend.

All these various effects of uterine disease sometimes require treatment, either in connection with the management of the radical difficulty, or after the lesion has been remedied.

The first, the most common and most persistent of the abnormal organic changes in the uterus, is that of engorgement. From this condition of the organ most of the lesions are supposed to take their origin. It is known by the increased size and weight of the uterus, as detected by digital examination, and by the feeling in the patient of weight—a heavy, dragging sensation in the pubic region when walking. Soreness on pressure, and in suddenly turning in bed, is felt by the patient, behind the pubis. This state of the organ becomes more and more fixed the longer it is suffered to remain, until the structure itself seems to be increased, amounting almost to ordinary hypertrophy. In the mean-time, however, various lesions are liable to be developed.

As this condition leads to functional disturbance, to the displacement of the organ, and often to structural lesions, its discovery and treatment at an early period are highly important. Investigation with the speculum is not generally necessary to determine the existence of engorgement. To one at all experienced in digital examinations, the increased size of the os and neck may be readily discovered, and in this an engorged uterus may be distinguished from one impregnated. The enlarged os becomes harder and appears firmer to the touch, in proportion to the length of time this state has existed. In addition to the sense of weight, and tenderness on pressure or sudden movement of the body, protracted and annoying pain is often felt in the womb, sometimes in the ovaria. The uterus, when in this condition, doubtless has greater specific gravity than when it has attained to the same size from pregnancy, owing to the thickening of its walls at the expense of the cavity in the former, and the compactness of its structure from engorgement with blood. While this difference in weight in the two states may not always be detected by examination, yet that it exists is reasonable to suppose, and also that the prolapsus so frequently found in an engorged womb, would very probably result.

That which would seem to be the most rational course of treatment, and that which experience has proved most successful, is local depletion. The necessary quantity of blood to be abstracted may be more readily and promptly obtained by leeching. Two or three leeches may be introduced through a speculum and applied to the os, which, with the hemorrhage that usually continues after their removal, will draw a sufficient amount of blood for the time. This may be repeated, if necessary, in one, two, or three weeks, as may seem to be required. Scarification, though not equal to leeching in many respects, may be of service. A few incisions in the edges of the os tincæ with a suitably shaped knife will, in some cases, afford sufficient amount of depletion to relieve, in a measure, some of the attendant difficulties.

Cups and blisters to the sacrum or hypogastric region are sometimes serviceable, from the counter irritation and slight local depletion which they effect, particularly when much tenderness and irritability exist.

Chronic or sub-acute inflamation of the whole substance of the uterus, or of particular parts, is a very common lesion of the organ. It is found to some extent in most cases of enlarged or engorged womb, but sometimes without such condition accompanying or preceding it. We have intimated that the whole or distinct parts of the womb may be the subject of chronic inflammation. Where the whole organ is affected, local depletion such as advised in engorgement, and counter-irritation by blisters, &c., are useful measures. When the os and neck alone are inflamed, scarification and the catheretic effect ot active astringents, such as sulph. copper, nit. silver, &c., generally give relief.

The most important, however, of these parts subject to the disease in question is the intra-uterine mucous membrane—important for two reasons: 1st. Because from its obscurity, when diseased it is very liable to be overlooked; and secondly, because of the difficulty met with in the application of suitable remedies. When affections of the womb are confined to this membrane alone, as is often the case, all that constitutional and local disturbance from reflex nervous impressions, in the forms of hysteria, dysmenorrhœa, &c., is kept up as in other forms of uterine disease; and yet, when uncomplicated, the speculum reveals no disease of the os or neck; nor do other modes of examination detect induration, or increase of weight or size. Under these circumstances, the practitioner is often perplexed in making his diagnosis and adopting the proper course of treatment.

From a more careful examination and enquiry, however, it will be found that there is a constant discharge of albuminoid viscid mucus from the os uteri. By close inspection, a thin, almost transparent fluid, resembling the white of an egg, will be seen as it flows slowly over the edge of the os. This is generally, though not always, in quantity sufficient to be noticed by the patient as a "slight leucorrhœa." From this symptom, the disease has been called "uterine catarrh;" but we prefer the term chronic *Endo-metritis*, which conveys to the mind a definite idea of the true pathological condition. The fallopian tubes and ovaria are often affected by the extension of the inflammation.

In the treatment of this, as well as all other chronic diseases of the uterus, very little permanent benefit results from the *elective* action of remedies, or what is called "constitutional treatment." Temporary relief from local pain, and the sometimes troublesome nervous disturbance, is afforded by the various neurotics; but, as in other local affections of a chronic character, have no other permanent effect than the establishment of a troublesome habit.

The local application of remedies is that upon which the only certain reliance can be placed for correcting this abnormal condition. Nitrate of silver, sulphate of copper, &c., may be used in the solid form or concentrated solution, with great benefit in this way. The difficulty of applying the remedy in the solid form to the whole interior of the uterus, is an embarrassing circumstance in the treatment; and the violent symptoms sometimes produced by the injection of liquid catheretics into the cavity justly deters the practitioner from this mode of procedure, ordinarily. Violent pain, called "uterine colic," is occasionally the result, and for this reason, though the most convenient and effective manner of application, is not generally practiced. A stick of lunar caustic, arranged in a suitable port caustic, may be readily introduced so as to come in contact with the internal surface of the neck, but cannot very well be made to reach other portions of the mucous membrane to any extent. The same difficulty is met with in the use of the probang with a sponge saturated with a strong solution of the nitrate of silver. Having the sponge made in a tapering form, however, and the end of a whalebone probang whittled down so as to be small and flexible, and let it penetrate the sponge longitudinally nearly its entire extent, the introduction may be effected when the cavity of the neck is of large size. Very little of the solution, however, reaches the surface of the body after being pressed through an ordinary sized canal.

For the introduction of remedies into the uterus, in any form, it is generally necessary that the os be dragged into and held in the speculum while the operation is made. A tenaculum, or forceps, with long slender shanks is necessary for this purpose.

The length of time between the applications should vary according to circumstances. Generally once a week the remedy should be applied; but, in some cases, once a month answers very well.

Another condition of the os uteri, about which a great deal has been said, is that of ulceration. In the opinion of the writer, however, this condition is very rarely met with. Slight abrasions are occasionally found when chronic inflammation of the os exists. Such do not have any characteristics of, nor can they be properly termed ulcers. The frequency with which this lesion is met with by some practitioners, while others rarely see it at all, is explained only by this common error in diagnosis. Ulcers are, however, sometimes found, penetrating even into the canal of the neck, and bleed upon the slightest touch. As they may be cauterized readily with the solid stick, they are generally cured by a few weekly applications of the nitrate of silver.

The embarrassing circumstances under which the practitioner labors in the treatment of uterine affections by the local application of remedies tend greatly to retard the cure. A correct diagnosis is not made in some instances until six months of pretended treatment have passed. A delicacy on the part of the practitioner, as well as the patient, often interferes with the proper investigation until the affection, though slight at first, has become extensive and permanent.

---

